# First person – Radoslaw Gora

**DOI:** 10.1242/dmm.049443

**Published:** 2022-02-24

## Abstract

First Person is a series of interviews with the first authors of a selection of papers published in Disease Models & Mechanisms, helping early-career researchers promote themselves alongside their papers. Radoslaw Gora is first author on ‘ Analysis of the H-Ras mobility pattern *in vivo* shows cellular heterogeneity inside epidermal tissue’, published in DMM. Radoslaw is a PhD student in the lab of Dr Marcel Schaaf at Leiden University, Leiden, The Netherlands, investigating protein mobility patterns by use of single-molecule microscopy techniques.


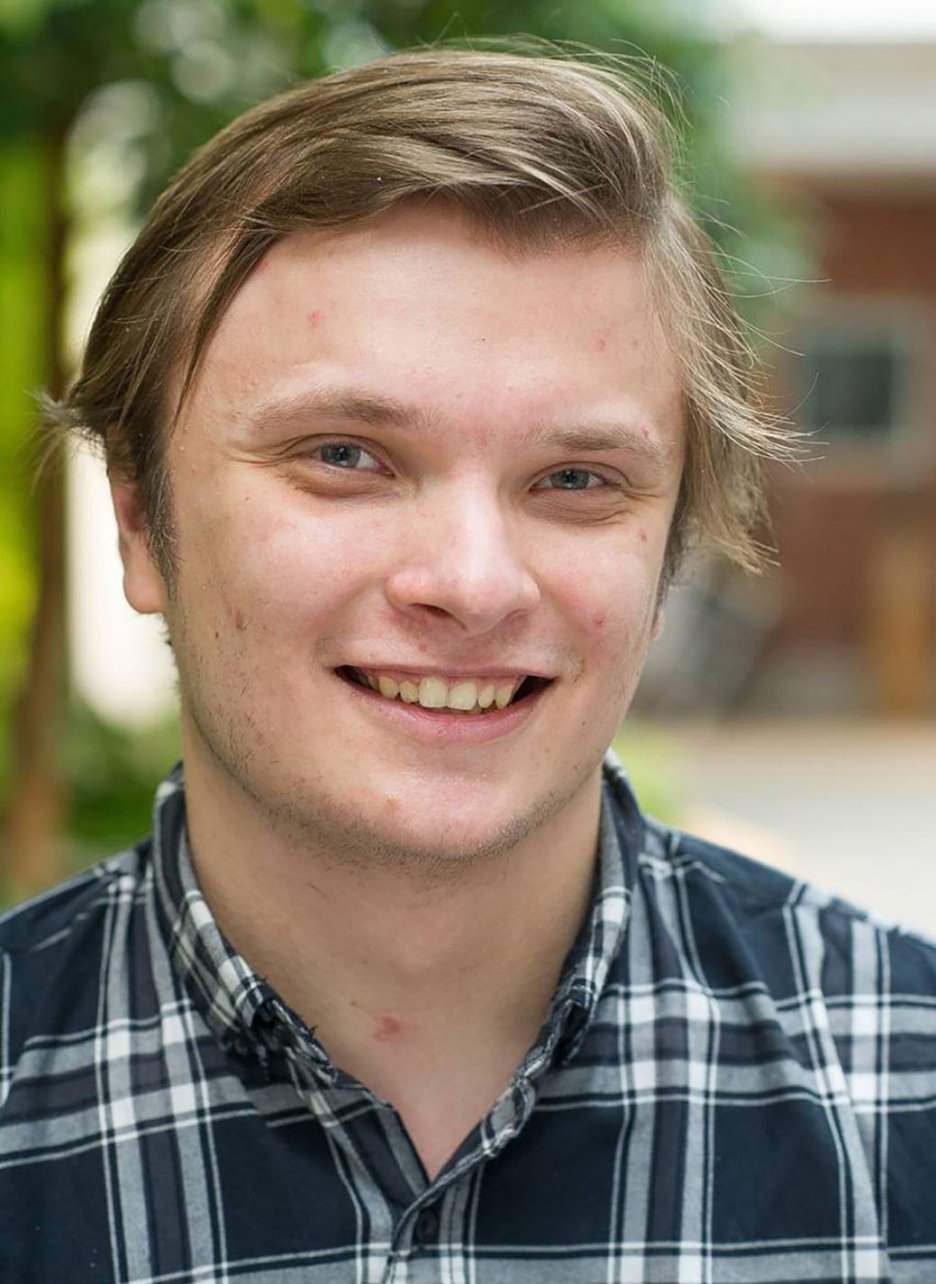


**Radoslaw Gora**

**How would you explain the main findings of your paper to non-scientific family and friends?**

Single molecules are in a constant move to process and transduce signals coming from different parts of the body. Organization and control of such a dynamic process requires sensitive and robust machinery so that every message is delivered to the correct address. In our studies, we focused on the mobility patterns of a membrane protein – H-Ras – that is attached to a cell membrane and carries the signal that ultimately tells the cell to grow and proliferate. As a biological model, we used a small, transparent vertebrate – the zebrafish – which, contrary to isolated cell cultures, tells us more about the dynamics of single proteins in an intact organism, reflecting all of its metabolic and signalling complexity. In the current study, we show that single H-Ras proteins belong to either of two fractions: one that diffuses fast and another that barely moves within the cell membrane. Interestingly, what has not been demonstrated in studies performed on cell cultures, we observe that single molecules in both of these fractions do not move within the entire surface of the cell membrane, but rather confine to small areas, as if they wanted to aggregate and enhance the power of the signal that must be delivered to the cell nucleus. While it explains the confinement of the slow-diffusing fraction, being in the range of 100-200 nm, the size of the confinement areas of the fast-diffusing fractions is approximately five times larger and, hence, must be controlled by different molecular mechanisms. In order to validate this hypothesis, we reduced the zebrafish cell membrane cholesterol levels and disrupted the actin filaments that are attached to the cell membrane. And indeed, these modifications significantly increased the diffusion rate and the confinement size of the fast-diffusing fraction, exhibiting that the dynamics of this subpopulation is controlled primarily by the structure of the cell membrane and not only by H-Ras genetic mutations, for instance. Finally, we characterized the sources of variability in our results and showed that most of the variability comes from imaging different cell areas, implying that it is the local environment of the zebrafish epidermal tissue that mostly defines the H-Ras mobility patterns.

**What are the potential implications of these results for your field of research?**

Our results build on the biophysical notion that the dynamics of the single molecules is a highly structured phenomenon, and that the same type of molecules might belong to different fractions, based on their diffusion rates and confinement area sizes. We demonstrate that the presence of different H-Ras subpopulations reflects their different activation states and, moreover, that the H-Ras mobility patterns are also controlled by structural components of the zebrafish epidermal cell membranes. In our paper, we aim to emphasize the importance of studying protein mobility patterns in addition to traditional biochemical approaches where the interactions between proteins are studied in order to reconstruct their signalling pathways. The findings that we report in the current paper might encourage other researchers to continue exploring the unknown world of the single-molecule dynamics. With a constant development of microscopy tools, it might soon be possible to determine whether single molecules indeed always belong to one of many subpopulations or rather exist in different dynamic states, depending on their transient biological roles or genetic mutations. Subsequently, with improving temporal and spatial resolution of the microscopic devices, scientists may better understand how often does a single molecule switch between its different molecular states and what are the factors that influence these processes. Such advancements in single-molecule microscopy studies might improve our knowledge on the role of the protein mobility patterns in a variety of major disorders, with the prime example being cancer growth and metastasis.

“The zebrafish is an extremely user-friendly model for single-molecule microscopy imaging.”

**What are the main advantages and drawbacks of the model system you have used as it relates to the disease you are investigating?**

The zebrafish is an extremely user-friendly model for single-molecule microscopy imaging. First of all, it is transparent, and any pigmentation might be further removed by chemical treatment with 1-phenyl 2-thiourea (PTU) or simply by the use of the zebrafish transgenic albino line. Transparency of this model allows for convenient imaging of any set of molecules, provided that they are fused to a fluorescent tag. Secondly, embryos develop outside of the uterus, which enables us to inject any DNA or mRNA construct at the one- or two-cell developmental stage, passing it on to the daughter cells of the growing larva. Thirdly, zebrafish embryos can be easily anaesthetized with tricaine, making them relatively easy to dechorionate, immobilize, mount and image.

With all its advantages, the zebrafish embryo is still a very fragile and delicate biological model. Its mounting and imaging require high precision, as any wrong movement of the tweezers might lead to damage to the entire embryo. Furthermore, zebrafish larvae cannot be imaged over the prolonged period of time, as the powerful excitation laser beams will, eventually, scar the sensitive tissues of the developing embryo.

**Figure:**
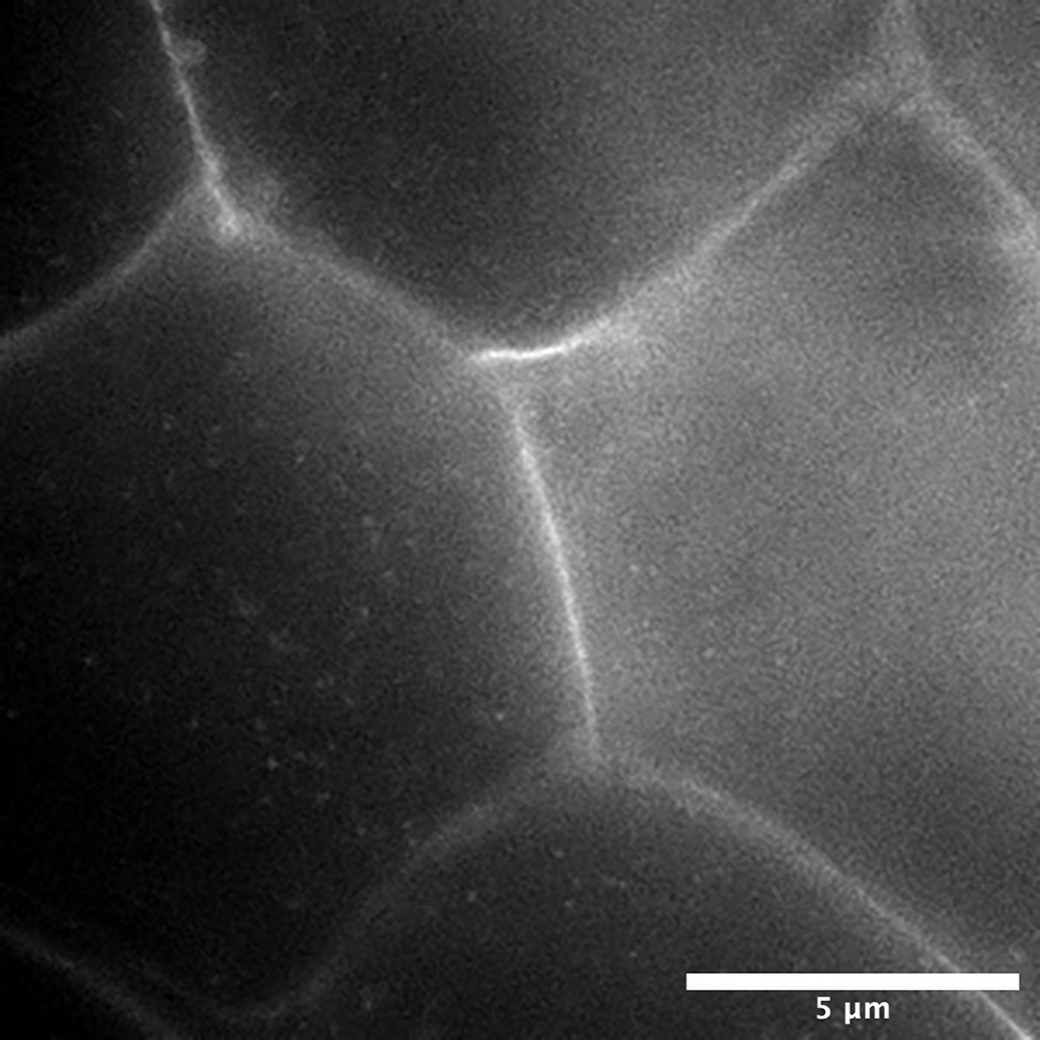
Localization of single H-Ras proteins inside epidermal cells of a living zebrafish embryo.

**What has surprised you the most while conducting your research?**

It has been surprising to see that zebrafish larvae can produce such repeatable results when it comes to the analysis of single protein mobility patterns. Taking into account the complexity of this model, when compared to cell cultures, we expected to see more variability in our results. Yet, after selecting four different time points of the larva development (from 48 to 80 h), we did not observe any significant changes in the diffusion rates and confinement area sizes of the H-Ras protein, indicating that the developmental stage of the zebrafish embryo does not define the H-Ras mobility patterns. Furthermore, after studying the sources of the overall variability in our results, we discovered that imaging different zebrafish embryos contributed the least to the total variability. This, in turn, implies that regardless of which embryo we image, we should observe very similar values of the dynamic parameters. We were extremely satisfied with these outcomes, as they make the zebrafish larvae a very robust model for *in vivo* single-molecule studies, and the results obtained by working with this model are reproducible and, therefore, verifiable.

**Describe what you think is the most significant challenge impacting your research at this time and how will this be addressed over the next 10 years?**

In the field of single-molecule microscopy, there are two major challenges that should be addressed over the next decade. Firstly, single molecules are practically nanoparticles that diffuse extremely fast within the cells of our bodies. To properly follow their molecular trajectories, spatial and temporal resolution of the microscopes must be further improved. We have already seen a rapid development of the super-resolution microscopy techniques, which, however, are not yet suitable for the *in vivo* single-molecule imaging, as their temporal resolution is not high enough and their image acquisition time is relatively long – too long for a zebrafish embryo to survive. Secondly, there is a need for better fluorophores, which rarely blink and their photobleaching capacities are significantly improved. With traditional fluorescent proteins, such as GFP or YFP, it is impossible to follow a single molecule over a long period of time, as they bleach after a few seconds. On the other hand, the synthetic dyes, such as Halo-tags, are not yet optimized for *in vivo* models and are very often simply washed away by the zebrafish larvae. Design of better fluorophores for *in vivo* applications will allow for easier reconstruction of the trajectories of the single molecules, without the need for extensive computational analyses and *in silico* modelling. Nonetheless, seeing the constant development in the fields of single-molecule microscopy, I am certain that in the next 10 years these obstacles will be overcome.

“Early-career scientists oftentimes find themselves in a very competitive environment, where they feel isolated and where they suffer from imposter syndrome.”

**What changes do you think could improve the professional lives of early-career scientists?**

Early-career scientists oftentimes find themselves in a very competitive environment, where they feel isolated and where they suffer from imposter syndrome. It is expected that they publish their experimental results, yet as it happens in the scientific field, their findings often do not match their corresponding hypotheses. I believe that, for the early-stage researchers, it is absolutely crucial to develop a healthy relationship with their supervisors. They should be able to communicate their worries, expectations and ideas without being judged by their superiors. Subsequently, they should try to develop certain personality traits, such as assertiveness and self-awareness, and learn how to set boundaries that should never be crossed. On the other hand, supervisors must understand that their students are a group of very different people who require personalized supervision techniques. It might often be that each of their students is motivated by a different type of feedback, and, in my opinion, a good supervisor should be sensitive enough to recognize these patterns and adapt to them. Otherwise, a lack of proper communication between the student and the supervisor might lead to a toxic professional relationship, full of discomfort, frustration and anger.

**What's next for you?**

I will most probably continue performing single-molecule microscopy studies as a postdoctoral researcher. I will certainly use my experience that I obtained during my PhD studies to investigate protein interactions or modes of nascent proteins membrane targeting on a single-molecule level. For future studies, I might employ different biological models, whether it be a synthetic lipid bilayer, cell cultures or an entire living organism, as I am not yet sure if I will have the opportunity to conduct my research on zebrafish embryos.
